# Micro-balloon-assisted embolization of anterior cranial fossa dural arteriovenous fistula via a trans-ophthalmic approach — a technical report and case series

**DOI:** 10.1007/s00234-022-02929-6

**Published:** 2022-03-21

**Authors:** Saminderjit Kular, George Tse, Bhavya Pahwa, Tony Goddard, Nayyar Saleem, Sanjoy Nagaraja, Richard Dyde, Tufail Patankar

**Affiliations:** 1 Department of Neuroradiology, Royal Hallamshire Hospital, Glossop Road, Sheffield, S10 2JF UK; 2 University College of Medical Sciences, Delhi, India; 3 Department of Neuroradiology, Leeds Teaching Hospitals, Leeds, UK

**Keywords:** Brain, Neurointervention, AVM, Embolization, Micro-balloon

## Abstract

**Purpose:**

Dural arteriovenous fistulas (dAVF) account for approximately 10–15% of all intracranial arteriovenous abnormalities. dAVFs carry a significant risk of mortality, particularly in cases of acute hemorrhage, of up to 10%. A small proportion of these dAVFs are found in the anterior cranial fossa (ACF), of which the rate of hemorrhage can be as high as up to 91%. The Scepter Mini (SM) is the smallest dual-lumen micro-balloon (MB) available for neurointerventional practice. It consists of a 2.8 French outer diameter, with a 2.2 mm × 9 mm semi-compliant balloon providing a working length of 165 cm. The SM is navigated with a 0.008-inch wire making it a particularly attractive tool accessible to the pedicles normally reached with liquid embolization micro-catheters.

**Methods:**

Five consecutive patients over a 1-year period between 2020 and 2021 were evaluated and treated for ACF dAVF using a liquid embolization approach using the SM balloon. All patients were treated using ethylene–vinyl alcohol copolymer (EVOH), of which Squid 18 and/or Squid 12 were the chosen viscosities. Control angiograms were performed for all patients post-embolization.

**Results:**

All patients demonstrated complete occlusion of the ACF dAVF on immediate post-treatment angiography. No immediate complications were encountered; particularly, there were no reports of visual field deficit in any of the patients.

**Conclusion:**

The MB is a valuable adjunctive tool that can enhance the safety and efficacy of trans-ophthalmic embolization of ACF dAVFs, providing additional protection to the retinal and posterior ciliary arteries against unwanted reflux of liquid embolic agent.

## Introduction

Dural arteriovenous fistulas (dAVF) account for approximately 10–15% of all intracranial arteriovenous abnormalities [1].

dAVFs carry a significant risk of mortality, particularly in cases of acute hemorrhage, of up to 10% [2].

A small proportion of these dAVFs, approximately 5.8%, are found in the anterior cranial fossa (ACF) [3]. Within these ACF dAVFs, the rate of massive intracranial hemorrhage can be as high as 62–91% [4, 5]. This is thought to be due to the preferential predilection for direct cortical venous drainage, consistent with a Cognard classification type 3 dAVF [6, 7]. This evolution of ACF dAVFs prompts urgent management, which can be performed via a surgical or endovascular approach in the acute setting. Radiosurgery is also an effective treatment option, however is utilized on an elective basis, rather than in cases of acute presentation [8].

Knowledge of endovascular treatment of ACF dAVFs is limited, with less than 50 cases having been formally published to date [5]. However, given recent advances in micro-catheter technology and embolization materials, endovascular treatment of ACF dAVF has increased and often advocated as the treatment of choice.

An important consideration of trans-arterial embolization of ACF dAVF is to avoid inadvertent embolization of the retinal artery, which can lead to subsequent debilitating blindness. Our case series describes the technical utilization of a dual-lumen micro-balloon (MB) (Scepter Mini — Microvention) to delineate the origin of the retinal artery and subsequently protect undesired reflux during embolization of the ACF dAVF.

**Fig. 1:**
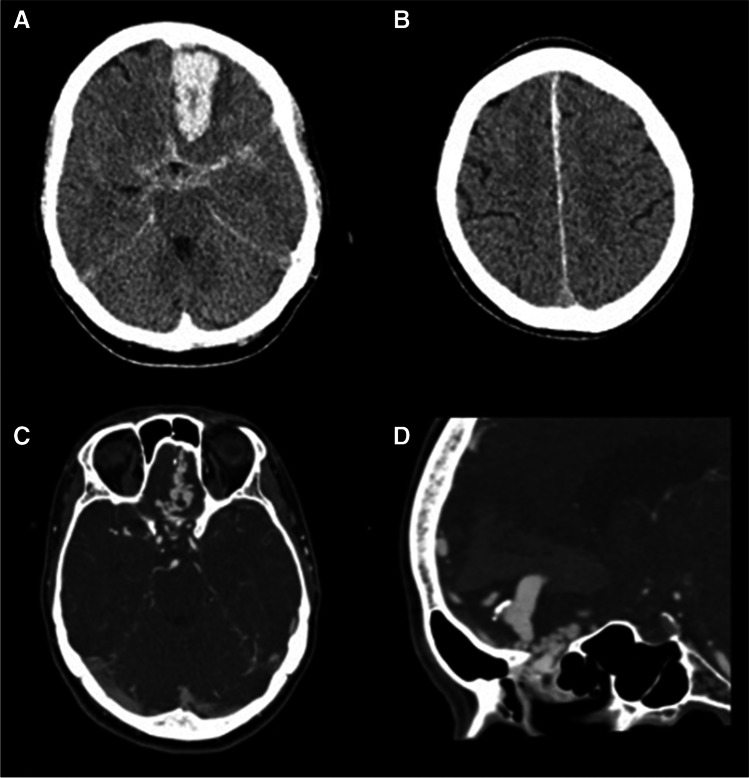
Selected case (patient case 5 from Table 1). **A** Sixty-three female presenting with sudden onset headache, nausea, and vomiting. Non-contrast CT head study demonstrated left frontal acute intraparenchymal hemorrhage with subarachnoid hemorrhage and **B** left parafalcine subdural hemorrhage. **C**, **D** Subsequent CT angiography (CTA) highlighted a network of several serpiginous underlying draining veins within the anterior cranial fossa

The Scepter Mini (SM) (Microvention, CA, USA) is the smallest dual-lumen balloon available for neurointerventional practice today (Fig. 1, adapted from [9]). It consists of a 2.8 French outer diameter, with a 2.2 mm × 9 mm semi-compliant balloon providing a working length of 165 cm (Fig. 1). The SM is navigated with a 0.008 inch wire [10].

This paper aims to outline our dual-center experience regarding liquid embolic agent (LEA) management of AVF dAVFs using adjunctive micro-balloon assistance.

## Methods and results

Five consecutive adult patients over a 1-year period between 2020 and 2021 were evaluated and treated for ACF dAVF using a liquid embolization approach (Table 1).

**Table 1:** Summary table of patient cohort. 5 patients were successfully treated with LEA. 3 patients presented acutely while 2 patients were incidentally detected on MRI

Patient	Patient age	Patient gender	Presenting complaint	Embolic agent	Result
1	79	Female	Incidental finding — post-fall	Squid 18	Complete occlusion
2	43	Male	Incidental finding — headache	Squid 12	Complete occlusion
3	60	Male	Collapse — acute intracranial hemorrhage	Squid 18 + 12	Complete occlusion
4	50	Female	Incidental finding — vestibular schwannoma follow-up scan	Squid 18	Complete occlusion
5	63	Female	Collapse — acute intracranial hemorrhage	Squid 18	Complete occlusion

**Fig. 2:**
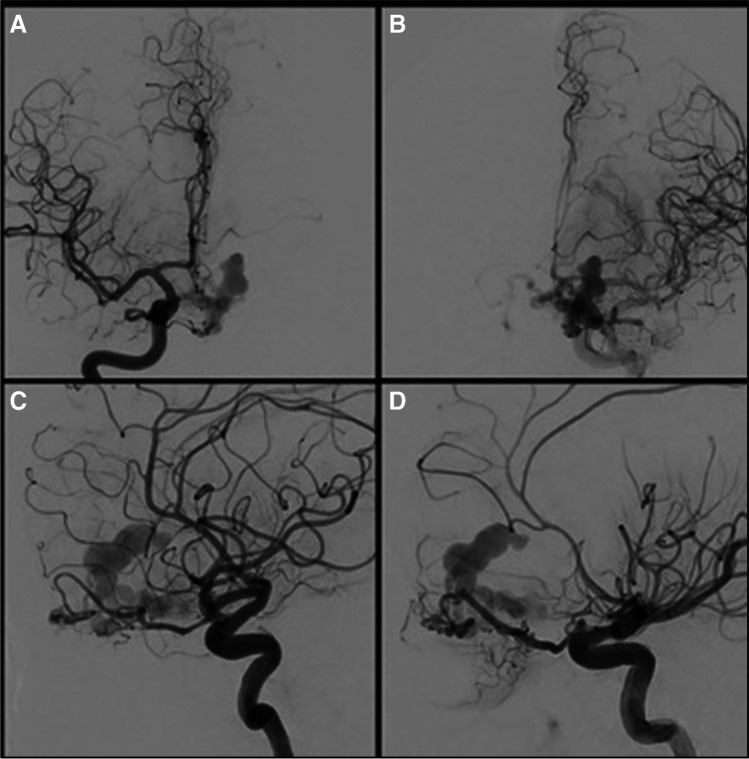
**A**–**D** Catheter angiography was performed, demonstrating a left frontal ACF dAVF, with a varix identified as the source of acute hemorrhage. Supply from both ophthalmic arteries was demonstrated with no significant external carotid artery supply

**Fig. 3:**
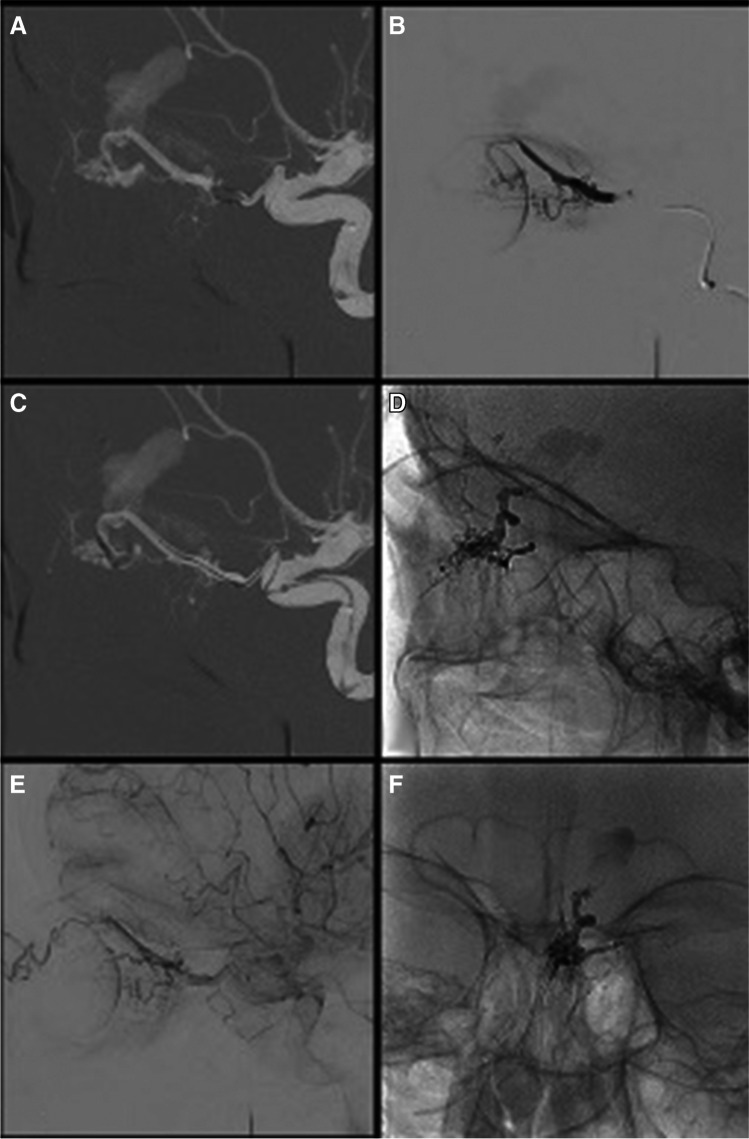
**A** Right ICA masked image for navigation of the MB. 8Fr NeuronMAX (Penumbra Inc.) in the left common carotid artery, DAC 0.044″ intermediate catheter (Stryker) navigated to the cavernous and ophthalmic ICA for support, particularly at origin of the ophthalmic artery. **B** Isolated ophthalmic artery angiography under balloon inflation identifying fistulous point and origin of the central retinal artery. **C** Distal navigation to the ethmoidal notch, note deformity as the balloon sits in the bone. **D**, **F** Final lateral and AP views of Squid cast. **E** Preservation of retinal blush with contrast stagnation in the hypertrophied ophthalmic artery, fistula, and shunt clearly obliterated

Three patients were discovered with acute intracranial hemorrhage on hospital-admission CT head and CT angiography (CTA) (example case in Figs. 2 and 3). Two patients were found to have incidental vascular abnormality on MRI, with subsequent MR angiography (MRA) being performed in these cases.

All patients had adjunctive LEA retinal protection using the SM micro-balloon. All patients were treated under general anesthesia using ethylene–vinyl alcohol copolymer (EVOH), of which Squid 18 and/or Squid 12 (Balt, Montmorency, Ile-de-France, France) were the chosen viscosities (Table 1). Control angiograms were performed for all patients post-embolization.

All patients demonstrated complete occlusion of the ACF dAVF on immediate post-treatment angiography. Extraction of the SM was entirely uncomplicated in all procedures with no evidence of balloon adhesion to the cast or vessel wall. No immediate complications were encountered, particularly no vessel wall injury or rupture. No anti-coagulation post-procedural medication was administered, nor were there any reports of post-procedural visual problems.

A 6-month check MRI/MRA was performed for assessment of disease residue or recurrence in all patients.

### Case example

An adult patient in their 60 s presented to the emergency department with a history of sudden onset headache, nausea, and vomiting. Subsequent CT head and CTA studies demonstrated acute intraparenchymal hemorrhage with an associated ACF-dAVF (Fig. 2). Swift angiographic transfer was arranged for LEA embolization.

An 8-Fr NeuronMAX 088 guide sheath was navigated to the left cervical ICA (Fig. 4A). A DAC 0.044 intermediate catheter (Stryker) was navigated to the petrous left ICA. Angiography visualized the ACF-dAVF (Fig. 3).

A 2.2 mm Scepter Mini dual-lumen MB and an Asahi Chikai 0.008 micro-wire combination were navigated into the left ophthalmic artery. The MB was inflated in a proximal position and micro-catheter contrast injection was performed (Fig. 4B). The origin of the left central retinal artery was clearly identified. The balloon was subsequently deflated and navigated as distally in the ophthalmic artery as possible, closer to the site of the ACF dAVF (Fig. 4C). The MB was then re-inflated and liquid embolization was performed with Squid 18 LEA. No LEA reflux was seen proximal to the MB.

Post-embolization angiography confirmed complete fistula occlusion (Fig. 4D/F). The left retinal blush was well maintained (Fig. 4E). The MB was subsequently removed, the patient made a full recovery without complication, and in particular no visual complication was observed.

## Discussion

A popular method of LEA embolization of vascular malformations is via the “pressure cooker” technique. This consists of creating a plug of coils and glue between the tip and the detachment zone of a previously placed dimethyl sulfoxide (DMSO)–compatible dual-lumen micro-catheter, in order to enable a continuous forward-flowing injection of LEA while avoiding concurrent reflux [11]. This method however is challenging for ACF dAVFs due to risk of unwanted ophthalmic artery embolization and is therefore best avoided in this scenario.

Open surgery may be a potential treatment option, which often produces an efficacious result [12]; however, it is inherently associated with other significant risks attributed to a more invasive and open procedure including cranial nerve palsy (particularly the olfactory nerve), infection, stroke, CSF leak, hydrocephalus, and severe blood loss [13]. In most cases, surgery is best reserved for when endovascular management cannot be achieved [8, 14].

A transvenous approach can also be used when undertaking endovascular LEA embolization of dAVFs. This is particularly useful in treatments of carotid-cavernous fistulas (CCFs), where there is a high probability of inadvertently embolizing dangerous anastomoses involving arteries that supply the cranial nerves [15]. Challenges involving a transvenous approach include an increased risk of cerebral hemorrhage, venous infarction, and vessel perforation due to reduced thickness of the media layer, of particular importance during micro-wire and micro-catheter manipulation [16]. There is also a greater distance the endovascular apparatus must travel if using a transvenous approach which in some instances can be a limiting factor.

LEA embolization using an adjunctive MB is a relatively new technique to treat ACF dAVFs, with only two patients being previously documented as having been treated via this approach [17]. Data regarding adjunctive MB embolization of dAVFs elsewhere within the brain is also scarce [17, 18], which is understandable given the short period of time the SM device has been commercially available for neuroendovascular use.

Regarding embolization of ACF dAVFs, injecting LEA via the ethmoidal artery is seen to be the preferential method of choice as the middle meningeal artery (MMA) can often be tortuous and difficult to access optimally [19].

The MB is of particular importance in treating ACF fistulas via a trans-ophthalmic approach, as reflux of LEA is a common phenomenon encountered in intracranial embolization. As a result of this, during a trans-ophthalmic approach, the more proximally located retinal and posterior ciliary arteries are at considerable risk of inadvertent embolization, potentially leading to disastrous patient blindness. In addition, we have demonstrated that isolated ophthalmic artery angiography under balloon inflation can identify the origin of the central retinal artery, again improving the safety of the procedure.

The MB can provide significant protection against reflux of LEA when sufficiently deployed distal to the retinal and posterior ciliary arteries, as close to the ACF fistula site as practical. Initial feedback from using the MB has been favorable, stating good navigability and flow arrest during several cases [17, 20, 21]. These positive factors were further supported by our experience. The MB can be additionally be used to provide superselective navigation and occlusion of the venous system in AVM embolization, particularly useful if access into the smaller and more distal cortical venous system is required.

While our two centers did not experience any notable drawbacks, some operators have noticed balloon kickback or “jump-back” and kinking of the balloon on inflation [20, 21]. Both of these factors, however, can be countered. For example, balloon kickback can be identified and avoided early if the operator is aware of it, helping to minimize any unwanted catheter movement with careful, controlled LEA injection and proactive observation. The single instance of documented balloon kinking occurred during attempted inflation within a tortuous segment of dural vessel and, on subsequent minor repositioning, inflated normally. This difficulty was relatively easily worked around and it should be noted that kinking within tortuous vessels is a problem not uncommon among all types of balloon and not specific to a MB.

Another limitation of our experience was the number of cases. As is the situation with all emerging technologies, our case numbers and the overall total number of cases within the literature is limited at present. However, cases that have been documented in the literature using a MB, in particular the SM, have been very positive, including those used for ACF dAVF embolization [17].

## Conclusion

The micro-balloon is a valuable adjunctive tool that can enhance the safety and efficacy of trans-ophthalmic embolization of anterior cranial fossa dural arteriovenous fistulas, providing additional protection to the retinal and posterior ciliary arteries against unwanted reflux of liquid embolic agent. Given their small size and ongoing future clinical uses, micro-balloons, including the Scepter Mini, will of no doubt be beneficial in facilitating the next frontier of neuroendovascular therapies, where navigation across small caliber intracranial vasculature is required.
